# Supplementary material for: Are carbon emissions trading and green financial instruments synergistic? -Comprehensive quantitative research based on content analysis
Source: PLoS One. 2024 Mar 7;19(3):e0298601. doi: 10.1371/journal.pone.0298601 (PMC10919601; doi:10.1371/journal.pone.0298601)
Supplement: S1 Data — (ZIP) [file pone.0298601.s001.zip › Data/地级市Z_F工作报告.zip!/╣π╢1⁄2/╖≡╔╜╩╨/└┤╫╘╣1⁄2╓┌║┼í╛┬φ┐╦╩2╛▌═°í┐_2019.docx]

各位代表：

现在，我代表市人民政府，向大会报告政府工作，请予审议，并请各位政协委员和其他列席人员提出意见。

一、2018年工作回顾

2018年是全面贯彻党的十九大精神的开局之年，是改革开放40周年，是决胜全面建成小康社会、实施“十三五”规划承上启下的关键一年。面对错综复杂的国内外形势和经济下行压力，市政府坚持以习近平新时代中国特色社会主义思想为指导，全面贯彻党的十九大精神，以习近平总书记重要讲话精神为统领，在省委、省政府和市委的正确领导下，在市人大、市政协的监督支持下，坚持稳中求进工作总基调，坚定践行新发展理念，统筹推进稳增长、促改革、调结构、惠民生、防风险各项工作，实现地区生产总值9935.88亿元，增长6.3%；地方一般公共预算收入703.14亿元，增长6.3%，全市经济社会保持平稳健康发展。

一年来，我们主要做了以下工作：

一是及时出台有效政策措施，促进经济运行保持在合理区间。出台落实“六稳”[]工作方案，多措并举应对经济下行压力。着力稳就业，就业形势保持稳定。城镇新增就业8.57万人，其中城镇失业人员再就业3.5万人，就业困难人员实现就业6768人，本市生源应届高校毕业生就业率95.1%，城镇登记失业率2.36%。省、市、区共建创业孵化示范基地投入运营。着力稳金融，金融服务实体经济能力提升。促进社会投资健康发展、企业债券发行、债券品种创新与风险防范等工作获国务院通报激励。金融机构存款余额1.54万亿元，增长9.5%；贷款余额1.05万亿元，增长11.5%，成为全省首个贷款余额突破万亿元的地级市；银行业机构不良贷款率为1%，下降0.46个百分点。新增上市企业5家，累计达58家；新增新三板挂牌企业6家，累计达92家；新增私募股权投资基金100家，累计达486家。着力稳外贸，外贸增长总体平稳。主动应对中美贸易摩擦影响，推动外贸调结构、拓市场，成为国家级市场采购贸易方式试点城市，完成进出口总额4599.3亿元，增长5.5%。其中，出口总额3527.4亿元，增长11.9%，对“一带一路”沿线国家出口1173.1亿元，增长9.7%。着力稳外资，外资利用水平稳步提高。优化外商投资环境，扩大对外开放投资领域，实际利用外资45.73亿元。引进粤维物流设备、维讯科技等超千万美元外资项目39个，合同外资11.29亿美元。着力稳投资，固定资产投资规模持续扩大。固定资产投资增长5.6%，其中工业技改投资增长8.5%。民间投资增长18%，占固定资产投资比重达75.1%。省、市重点项目投资891.24亿元，完成全年进度目标。着力稳预期，提振民营企业发展信心。召开民营企业家大会，出台促进民营经济高质量发展“1+3”政策文件[]。降低生产要素成本及制度性交易成本，为企业减负426.02亿元。深入开展“暖企”行动，帮助企业解决用地、用工、用电等实际问题234项。民营经济增加值占地区生产总值比重达62.5%，规模以上民营工业增加值增长7.1%，对全市工业增长贡献率80.1%。新登记市场主体15.72万户，增长30.9%，市场主体总数达72.03万户。扶持骨干企业做强做大，新增主营业务收入超100亿元企业2家、累计达20家，14家企业入选省百强民营企业。实施小微企业上规模专项行动，认定“专精特新”企业306家。培育发展“四上”企业，入库企业净增1500家，总数达1.36万家。

二是深入推进供给侧结构性改革，制造业转型升级取得新成效。国家制造业转型升级综合改革试点扎实推进，工业稳增长和转型升级成效较明显，获国务院通报表扬。三次产业比重调整为1.5：56.5：42，规模以上工业增加值增长6.3%，工业对经济增长贡献率达61.1%，服务业占比提高1.1个百分点。工业经济质量效益稳步提高，规模以上工业经济效益综合指数311.87%，提高14.4个百分点，利润总额增长5.9%。传统产业改造升级力度加大。引导1224家规模以上工业企业开展技术改造和智能化高端化改造，新增省级智能制造试点示范项目28家、市级智能化改造示范企业32家，推广应用机器人3014台。促进制造业与互联网融合发展，新增27家国家和省级“两化”融合管理体系贯标试点企业。规模以上装备制造业增加值增长6.4%，其中“工作母机”增长7.2%。培育发展新兴产业步伐加快。规模以上先进制造业增加值增长7.4%，占规模以上工业比重49%，提高2.4个百分点。新签约投资超亿元内资项目462个，计划投资3039亿元。投资800亿元的碧桂园“机器人谷”、150亿元的航天军民融合协同创新智慧城等项目签约，投资118亿元的一汽—大众新能源汽车、90亿元的中国中药总部、60亿元的美的库卡智能科技园、20亿元的大疆创新科技等项目开工，科力远混合动力项目一期建成投产。制造业品质革命深入开展。积极创建全国质量强市示范城市，工业产品质量监督抽查综合合格率94.6%，提高1.1个百分点。新认定细分行业龙头企业216家，累计达370家；新增质量管理体系认证企业1543家，累计达7575家。推动企业参与制（修）订国际、国家和行业标准100项，累计达1769项；制定实施联盟标准36项，累计达225项。获批创建全国知名品牌示范区4个、累计达13个，驰名商标总量160件，均位居全国地级市首位。新增广东省名牌产品268个，累计达580个，总量位居全省首位。109家企业入选广东制造业500强，增加51家。服务业发展提质增效。现代服务业增加值增长7.6%，占第三产业比重59.8%，提高0.3个百分点。高端服务业载体建设成效明显，广东金融高新区集聚银行、证券、私募创投等项目523个，总投资达1010亿元；千灯湖创投小镇一期基本建成，新引进股权投资基金152家，资金规模124亿元；广东工业设计城聚集设计企业253家、研发设计人员8195名。会展业水平持续提升，成功举办第四届珠江西岸先进装备制造业投资贸易洽谈会、第四届中国（广东）国际“互联网+”博览会、第十届国际发明展、中国安全产业大会等重大展会活动。全域旅游发展格局加快形成，旅游业总收入809.14亿元，增长14%；接待游客5424万人次，增长10%，其中过夜游客1695万人次，增长13.2%。启动“粤菜师傅”工程，成立全省首家厨师学院。南方影视中心加快建设，影视制作企业达467家。消费市场规模不断扩大，社会消费品零售总额增长8.9%。创建全省首个“放心消费”试点城市，消费环境评分位居全省首位，城市消费者满意度位居全国第4位[]，居民消费价格涨幅为2%。

三是深入实施创新驱动发展战略，科技创新水平能力得到新提升。获批建设国家创新型城市，财政科技投入54.65亿元，预计研发经费支出占地区生产总值比重2.42%。研发创新能力持续增强。季华实验室主体工程动工，引进新加坡半导体仪器等研发团队。清华大学佛山先进制造研究院揭牌，中科院苏州纳米所佛山研究院、佛山（华南）新材料研究院、清华大学城市安全研究中心、省科学院佛山产业技术研究院等新型研发机构落户。开展高新技术企业树标提质行动，新增国家高新技术企业1350家、累计达3900家，认定规模以上标杆高新技术企业50家。规模以上工业企业研发机构建有率51%；新增省级企业重点实验室5家，累计达26家；新增省级工程中心83家，累计达711家。建有科技企业孵化器85家、众创空间62家。区域创新平台建设提速。推进珠三角国家自主创新示范区建设，出台“一环创新圈”战略规划和三龙湾高端创新集聚区综合规划，佛山国家高新区全国排名升至第25位。佛山军民融合创新示范区核心区启动建设，张槎街道获批创建全省首个军民融合科技产业创新试点镇。创新创业环境进一步优化。出台“人才新政23条”，新引进领军人才超过50人，新增省级创新创业团队1个、市级创新创业团队48个。推进佛山科学技术学院创建高水平理工科大学，与德国亚琛工业大学合作筹建佛山理工大学，北京科技大学顺德研究生院、北京外国语大学佛山研究生院正式招生。成立全省首个技工教育集团，启动实施技能人才“2357”工程[]。中国（佛山）知识产权保护中心正式运营，专利申请量增长27.9%，授权量增长38.7%。加快科技成果转移转化，技术合同成交额7.46亿元，增长179%。

四是坚持深化改革开放，经济发展动力活力持续增强。以实际行动庆祝改革开放40周年，多项改革开放举措走在全省乃至全国前列。改革攻坚力度加大。积极探索构建推动高质量发展体制机制，顺德区获批率先建设全省高质量发展体制机制改革创新实验区。深入实施“放管服”改革，加大简政放权力度，取消和调整218项权责事项。开展“减证便民”事项清理，取消132项证明事项。在全国率先实施“3+5”商事制度改革模式[]和启用全程电子化“零见面”24小时智能商事登记系统，成为全国“企业开办全程网上办”改革试点城市，在全省率先复制“证照分离”改革试点经验。“一门式一网式”政府服务改革获“中国法治政府奖”[]，“区块链+共享社区”入选中国“互联网+政务”优秀实践案例[]。成为全省“信用奖惩、一键搞定”试点城市。探索科学划分市、区、镇（街）管理职能，研究出台加强市级财政统筹工作方案。稳步推进机构改革，深化事业单位分类改革。深化国资国企改革，新增混合所有制企业15家。农村土地承包经营权确权登记颁证基本完成，南海区中央农村土地制度改革试点取得阶段性成果，顺德区全国农村集体产权改革试点通过验收。开放水平全面提升。积极参与“一带一路”建设。举办中国—以色列跨境投资大会等国际交流活动，在美国、南非、坦桑尼亚设立佛山泛家居品牌产品海外展示体验馆。全方位加强对外交往。荣获国际友好城市交流合作奖[]，德国总统等多国政要到访，德国因戈尔施塔特市、日本伊丹市等友城代表团来访交流。中德工业城市联盟成员及观察员城市增至41个，其中德国城市18个。举办第三届“香港·佛山节”等活动，深化与粤港澳大湾区城市及泛珠区域高铁经济带城市合作发展，推动成立粤桂黔高铁经济带高新区协同创新联盟。广佛同城化深入推进，加强创新驱动发展战略合作，共建广佛科技创新产业示范区，广佛放射线二期、番海大桥等项目启动建设，1396个政务事项实现“异地申请、跨城通办”。

五是扎实推进城乡统筹发展，城市治理取得新成绩。城市治理三年行动计划稳步推进，968个项目已完工374个、开工460个，累计完成投资2580亿元。城市形态功能品质优化提升。实施中心城区城市形态提升三年专项行动，已开工28个项目，开工率58%，完成投资116.67亿元。出台产业发展保护区划定及城市棕线管理办法，保障350平方公里产业用地规模。加强城市更新（“三旧”改造）和村级工业园整治提升，新增“三旧”改造实施项目155个，占地面积1.59万亩，已完成106个，占地面积1.05万亩，投入资金260.93亿元。治理违法建设1596.93万平方米。特色小镇建设成效初显，岭南文荟小镇、仙湖氢谷小镇等12个小镇入选省级特色小镇创建名单，数量居全省首位。首批市级特色小镇256个重点项目已开工192个，完成投资271.35亿元。现代化基础设施建设稳步推进。珠三角枢纽（广州新）机场前期工作启动，地铁2号线一期、3号线、广州地铁7号线西延顺德段建设顺利，广佛地铁全线开通。“一环”高速化主线改造基本完成，“一环”西拓工程加快推进，新打通“断头路”20条。新建公交枢纽9个，中心城区公交专用道达157.2公里，新能源和清洁能源公交车比例达100%，公交车实现移动支付全覆盖。累计建成充电站120座、充电桩4000个、加氢站5座、地下综合管廊42公里，海绵城市达标面积53.23平方公里。新增光纤用户39万户，基本实现4G城乡全覆盖。220千伏坡丹甲乙线等9项重点电网工程建成投产。乡村振兴战略全面实施。出台乡村振兴“1+7+X”政策体系[]，启动高明革命老区特别帮扶计划，三水区成为省乡村振兴综合改革试点。推进6个省级新农村连片示范工程，完成农村人居环境整治和“五好”新村居建设任务。深化农业供给侧结构性改革，与省农科院共建农业科技示范市，新增市级现代农业园区5家，累计达50家；新增省级农业龙头企业5家，累计达52家；新增市级菜篮子基地9家，累计达63家；“三品一标一名牌”[]认证产品累计达251个。

六是坚决打好三大攻坚战，三年行动开局良好。积极有效防范化解各类风险。加强地方政府债务管理，消化地方政府债务160.56亿元，降幅11.9%。防范化解金融风险，打击违法金融活动。严格落实房地产调控政策，有效化解轮候网签风险，房地产市场保持平稳健康运行。持续改善生态环境质量。单位地区生产总值能耗、主要污染物排放量下降幅度完成省下达的目标任务。完成70家省控重点VOCs[]企业综合整治，淘汰禁燃区高污染燃料设备205台，二氧化硫、二氧化氮、PM2.5、PM10及臭氧浓度齐齐下降，其中PM2.5首次达到国家空气质量二级标准，全年空气优良天数超八成。落实河长制和湖长制，投入50亿元推进广佛跨界河流整治项目160个，饮用水源水质保持100%达标，11个国控、省控考核断面水质达到省考核要求。完成44间污水处理厂提标改造，新建（改造）污水管网304.82公里。认真落实中央环保督察“回头看”以及省环境保护督察整改，率先开展市级生态环境督察，完成水源地环境问题专项整治任务，完成村级工业园6027家企业环境整治，基本完成100项环保民生实事。划定生态控制线面积1951.53平方公里、城市蓝线面积562.6平方公里。创新实行岛长制，开展48个河心岛生态修复工作。大湾区高品质森林城市建设初见成效，新增绿化面积3.27万亩，市域森林覆盖率37.4%、建成区绿化覆盖率44.3％，新增（改造）公园绿地318.7公顷、绿色建筑1098.34万平方米。顺德区获评国家生态文明建设示范区，顺控环投热电项目投入运营。深入推进对口帮扶。积极开展新时期精准扶贫对口帮扶云浮、湛江工作，投入财政资金13.91亿元，所帮扶村农村居民人均可支配收入水平达到省考核要求，佛山（云浮）产业转移工业园创建国家级氢能技术标准创新基地。深入开展东西部扶贫协作，落实财政资金9.86亿元，对口扶贫协作四川凉山州工作在全国考核中获评“好”等次，对口支援西藏墨脱县、新疆伽师县、新疆生产建设兵团41团草湖产业园以及四川甘孜州乡城县、得荣县，对口合作黑龙江双鸭山市工作扎实推进。

七是加快发展社会民生事业，人民生活水平稳步提高。居民人均可支配收入4.96万元，增长8.3%。财政民生支出612.64亿元，增长6.8%，占一般公共预算支出75.9%，省、市民生实事全面完成。民生福祉持续改善。最低生活保障标准提高至每人每月980元，特困人员供养标准提高至平均每人每月1863元，孤儿养育标准提高至每人每月2000元，市受助人员托养中心建成投入使用。企业职工养老保险人均养老金提高至每人每月3200元，城乡居民养老保险基础养老金提高至每人每月220元，实现全市城乡居民养老保险全覆盖。随军家属一次性安置补助金标准提高至10.9万元，优抚对象抚恤补助标准最高增幅达32%。政府投资建设的公租房累计分配19900套，分配率96.9%。住房租赁监管及交易平台上线，新增上线房源31356套，签约6530套。公共服务水平不断提升。完成新（改、扩）建幼儿园43所，新增学前教育学位1.7万个，普惠性幼儿园占比达76%。新（改、扩）建义务教育阶段学校41所，新增学位4.2万个。推进省现代职业教育综合改革示范市建设，现代学徒制试点通过验收。实施“登峰计划”，启动11家高水平医院建设，积极打造珠江西岸医疗高地。国家卫生城市复审通过复查，209万居民签定家庭医生服务协议，为超过10万名新生儿提供常见遗传代谢疾病免费筛查服务。实施药品和医用耗材供应链改革，药品采购价格降低11.2%，医用耗材采购价格降低21.8%。新增社区居家养老设施88家，总数达332家；新建收养性床位3827张，总数达3.7万张。构建政社互动型复退军人服务体系，建成各级复退军人服务组织803个。优化新市民服务，实施新市民业务办理“最多跑一次”制度。创建成为国家公共文化服务体系示范区，佛山大剧院和国际体育文化演艺馆投入使用。开展50公里徒步、广东（佛山）非遗周暨佛山秋色巡游等活动，成功举办第27届中国金鸡百花电影节。启动社会综合治理云平台，获评全国社会治理创新示范市[]。加强精神文明建设，实施“乐善之城”建设三年行动计划。深入推进“厕所革命”，新（改）建各类公共厕所322个。平安佛山建设扎实推进。开展扫黑除恶专项斗争，创建全国禁毒示范城市，刑事警情下降26.9%，治安警情下降16.8%，社会治安持续好转。创建国家食品安全示范城市中期绩效评估获评最优等次，“明厨亮灶”餐饮单位达3.25万家，认定餐饮服务食品安全A级单位1048家、食品安全示范点1172家。积极稳妥做好非洲猪瘟防控工作。粮食安全责任省考核连续两年获优秀等次。落实安全生产“一岗双责”，商贸制造业、火灾、道路交通实现事故总量、伤亡人数“双降”目标。

八是坚持依法行政，人民满意政府建设取得新进展。坚持和加强党的领导，认真学习贯彻习近平总书记重要讲话精神，积极开展“大学习、深调研、真落实”活动，把全面从严治党贯穿到政府工作各方面。主动向市人大及其常委会报告工作，向市政协通报情况，依法接受监督，办理人大代表建议188件、政协提案215件，办复率100%。提请市人大常委会审议法规草案2件、制定修改政府规章3项，在全省依法行政考评中获优秀等级。建设人民满意政府完成115个年度重点项目，第三方机构评估得分87.25分。推进“数字政府”建设，获中国智慧城市建设领先奖和中国营商环境创新奖。自觉接受巡视监督和监察委员会的监督，审计监督全覆盖深入推进。加强党风廉政建设，严格落实“一岗双责”，驰而不息正风肃纪，政府作风持续改善。

各位代表，过去一年的成绩来之不易，这是习近平新时代中国特色社会主义思想科学指导的结果，是省委、省政府和市委坚强领导的结果，是全市人民凝心聚力、团结拼搏的结果。在此，我代表市人民政府，向全市人民，向各位人大代表和政协委员，向各民主党派、各人民团体和社会各界人士，向中央、省驻佛山有关单位，向驻佛山部队官兵和消防救援队伍指战员，向关心和支持佛山改革发展的港澳台同胞、海外侨胞和国际友人，表示衷心的感谢！

同时，我们也清醒地认识到，我市经济社会发展不平衡不充分的问题依然存在，政府工作还存在诸多不足：一是经济下行压力增大，发展质量效益有待提高，传统产业转型升级任重道远，新兴产业还未形成有力支撑，重大创新平台、创新型龙头企业和高端创新人才缺乏，科技研发投入相对不足，新旧动能接续转换有待提速；二是城乡区域发展不平衡，城市形态功能品质与经济发展水平不匹配，基础设施建设欠账较多，一些重点项目工程推进较慢，村级工业园整治提升任务繁重，城市管理不够精细科学，智能交通服务能力有待提升；三是生态环境保护压力依然较大，大气、水、土壤环境污染问题未能根本解决，绿色生产生活方式尚未形成；四是社会治理能力有待提升，安全生产形势仍然严峻，教育、医疗、养老、文化等优质公共服务水平与人民群众期待还有较大差距；五是政府执行力、行政效能需进一步提升，少数公职人员干事创业热情有所减退，存在不想为、不敢为等现象。我们将直面问题，勇于担当，在今后工作中认真加以解决。

二、2019年工作安排

2019年是新中国成立70周年，是决胜全面建成小康社会第一个百年奋斗目标的关键之年，做好今年工作至关重要。当前国际形势复杂多变，世界经济周期呈现整体性回落趋势，中美经贸摩擦是我国外部发展环境中最大的不确定性因素，国内经济运行稳中有变、变中有忧，经济面临下行压力，这些都给佛山今年的发展带来挑战和不确定性。但同时，我国发展仍处于并将长期处于重要战略机遇期，经济正由高速增长阶段转向高质量发展阶段。我市面临中央和省大力支持实体经济和民营经济发展、“一带一路”建设、粤港澳大湾区建设及“一核一带一区”发展新格局建设等重大机遇；我市经过改革开放40年发展，积累了较雄厚的经济基础和综合实力，拥有经济结构优化升级、制造业提质增效、民营经济保持活力、营商环境优良等发展优势。我们必须坚定信心、迎难而上，化挑战为机遇，变压力为动力，以闻鸡起舞、日夜兼程，风雨无阻、勇往直前的精神状态，扎实做好全年各项工作，推动经济社会发展取得新成绩。

今年政府工作的总体要求是：高举习近平新时代中国特色社会主义思想伟大旗帜，深入学习贯彻党的十九大和十九届二中、三中全会以及中央经济工作会议精神，深入学习贯彻习近平总书记对广东重要讲话和一系列重要指示精神，按照省委、省政府决策部署和市委十二届七次全会部署，统筹推进“五位一体”总体布局，协调推进“四个全面”战略布局，坚持稳中求进工作总基调，坚持新发展理念，坚持推动高质量发展，坚持以供给侧结构性改革为主线，继续用好改革开放“关键一招”，牢牢扭住推进粤港澳大湾区建设这个“纲”，统筹推进稳增长、促改革、调结构、惠民生、防风险、保稳定各项工作，进一步稳就业、稳金融、稳外贸、稳外资、稳投资、稳预期，打好三大攻坚战，深入落实省委“1+1+9”工作部署[]，突出“抓落实、求突破”，保持经济持续健康发展和社会大局稳定，为全面建成小康社会收官打下决定性基础，以优异成绩庆祝中华人民共和国成立70周年。

今年经济社会发展主要预期目标是：地区生产总值增长6%-6.5%；全社会固定资产投资增长9%；社会消费品零售总额增长9%；进出口总额增长3%；地方一般公共预算收入增长6%-6.5%；研发经费支出占地区生产总值比重2.5%左右；居民消费价格涨幅控制在3%以内；居民人均可支配收入增长与经济增长基本同步；城镇登记失业率控制在3.5%以内；单位地区生产总值能耗、主要污染物排放量下降幅度完成省下达的目标任务。

围绕以上主要目标，今年重点做好以下工作：

一、全力以赴稳增长，精准发力推动经济平稳健康发展。坚持稳中求进工作总基调，突出在保持战略定力、增强企业活力、释放内需动力、增强经济发展内生动力上精准发力，着力稳住经济运行，以合理增速为提升发展质量创造条件。

推动工业经济运行稳中向好。发挥重大工业项目引领支撑作用，实行市、区领导挂钩联系制度，投资额50亿元以上的由市主要领导挂钩联系，20亿元以上的由市领导挂钩联系，10亿元以上的由区领导和市直部门领导挂钩联系，加快推进碧桂园“机器人谷”、一汽—大众新能源汽车、美的库卡智能科技园、大疆创新科技、乐华家居总部生产基地、织梦小镇等重大项目建设，促进在建项目尽快释放产能。持续开展“暖企”行动，帮助企业解决用工、用地、融资等实际困难。扎实开展第四次全国经济普查工作，加强普查数据分析运用，为推动经济行稳致远和科学决策夯实数据基础。

狠抓固定资产投资。加大轨道交通、公路、环保基础设施等投资力度，加强人工智能、工业互联网、物联网等新型基础设施建设，推动基础设施和公共服务设施向农村延伸。加大招商引资力度，完善“大招商、招大商”工作机制，突出项目亩均效益和科技含量，瞄准电子信息、机器人及智能装备、新能源、新材料等新兴产业精准招商，以重大产业项目建设带动工业投资。实施工业企业技术改造三年行动计划，放宽技术改造事后奖补范围，引导1200家以上工业企业开展技术改造。加强教育、医疗、养老等民生领域投资，加大学前教育及义务教育学位建设投入，加快市第二人民医院新院、三水新城医院等项目建设。拓宽民间投资领域，打破阻碍民间投资的“玻璃门”“弹簧门”“旋转门”，切实增强民间投资活力动力。

积极扩大消费需求。适应物质产品和服务消费升级趋势，积极培育消费新增长点，多渠道扩大服务供给、提升产品质量、改善消费环境，让市民群众吃得放心、穿得称心、用得舒心。推动实体零售创新发展，引导专业市场转型升级和商业综合体差异化发展，提高电子商务发展水平，促进线上线下融合消费。积极创建“放心消费”城市，加强消费环境安全和维权体系建设，培育一批示范性放心消费商家和企业。推动全域旅游扩面提质，拓展工业旅游、岭南民俗文化旅游、美食旅游、航空旅游、乡村旅游等精品旅游产品，完善各类旅游设施和服务体系，打造全国知名旅游目的地。实施“粤菜师傅”工程，打造优质粤菜食材基地，到2020年成立1家省级和20家市、区级粤菜师傅培训基地，培养粤菜师傅3000人以上，各区各打造1-2条特色美食文化街区。

促进外贸外资稳定增长。主动应对国际经贸形势变化，推动出口市场多元化，优化出口产品结构，扩大一般贸易基础。加快培育贸易新业态，推进亚洲国际家具材料交易中心市场采购贸易方式试点，积极申报佛山综合保税区，大力发展跨境电子商务，支持外贸综合服务企业发展壮大。加强出口质量安全示范区建设，培育出口名牌企业，促进产品“同线同标同质”。加强冻品、乳制品、水产品等进口商品集散地建设，扩大先进技术、关键设备、零部件和稀缺资源型产品进口规模。实施扩大对外开放实现利用外资高质量发展若干政策措施，提升外商投资贸易便利化水平，吸引更多优质外资项目落户佛山。

二、坚持以供给侧结构性改革为主线不动摇，推动制造业高质量发展。把发展经济的着力点放在实体经济上，在“巩固、增强、提升、畅通”上下功夫，提高供给体系质量和效率，加快建设具有国际竞争力的现代产业体系。

大力培育发展先进制造业集群。加快发展通用装备、专用装备、环保装备等装备制造产业，提升家电、家具、铝型材、纺织等家居产业发展水平，推动传统产业高级化、新兴产业高端化，力争到2020年培育形成装备制造、家居2个超万亿先进制造业产业集群，到2025年培育形成汽车及新能源、军民融合及电子信息2个超5000亿元产业集群，智能制造装备及机器人、新材料、食品饮料、生物医药及大健康4个超3000亿元产业集群。

巩固“三去一降一补”成果。深入推进国家制造业转型升级综合改革试点，探索完善促进制造业转型升级的财税支持、金融服务、技术创新、贸易投资、要素供给、市场监管等政策体系。加大“僵尸企业”退出力度，破除无效低效供给，加快推进村级工业园综合整治，到2020年完成23.06平方公里村级工业园整治提升。落实国家大幅度减税降费政策措施和省、市“降成本十条”，完善工业用地弹性出让、工商业用电用气价格定价、工伤保险费率浮动调整等政策机制，推广“众陶联”“众塑联”等市场化降成本模式，合力促进实体经济降本增效提质发展。围绕加强基础设施建设、营商环境建设等关键领域补短板。

着力增强企业发展活力。全面落实中央、省、市促进民营经济发展政策措施体系，形成政策叠加效应。扶持民营企业梯队成长，推动中小微企业“个转企、小升规、规改股、股上市”，深化大型骨干企业培育行动，培育一批“专精特新”隐形冠军，力争净增“四上”企业2000家以上，其中净增规模以上工业企业1500家，年主营业务收入超百亿元企业达22家、超50亿元企业达36家，形成大企业顶天立地、小企业遍地珍珠的产业协作生态体系。依法平等保护民营企业和企业家权益，严格区分“六个界限”[]，严肃查处侵权行为，重点保护民营企业家的人身权和财产权。弘扬企业家精神，表彰“脊梁企业”“大城企业家”，让民营企业家安心经营、放心投资、专心创业。

加快提升产业链水平。加大产业强链、补链力度，提升佛山制造在全球产业链、供应链、价值链中的地位。着力引进一批产业带动力强、投资额大的上下游关联项目，力争每年新增世界500强、行业龙头企业投资大项目10个以上。以智能制造为主攻方向推动传统产业优化提升。加快建设省智能制造创新示范园，打造一批智能制造示范企业以及重点行业机器人应用示范项目，新增智能化技改示范企业32家、机器人应用3000台。推进工业互联网广泛应用，支持企业依托工业互联网平台加快数字化、网络化、智能化发展，到2020年实现2万家企业“上云上平台”。继续推进佛山制造品质革命，创建全国质量强市示范城市，完成工业产品质量提升三年行动计划，参与“百城千业万企对标达标提升专项行动”，努力把佛山制造打造成为中国制造最高品质。推进“标准领跑者”工作，引导企业主导或参与国际、国家、行业标准制（修）订。强化个体品牌和区域品牌建设，确保全市获批创建知名品牌示范区占主导优势产业集群比例达95％以上。

畅通金融服务实体经济渠道。在信贷、债券、股权等方面精准发力，切实缓解企业融资难融资贵问题。搭建金融信用服务平台，完善中小微企业信用信息和融资对接平台，为企业融资提供精准服务。支持银行扩大信贷投放，健全信贷风险损失补偿机制，成立融资担保专项基金，拓宽支持企业融资专项资金覆盖面。加大债券融资支持力度，灵活运用债券融资风险缓释基金和民营企业债券融资支持工具，引导企业通过债券市场融资。设立融资租赁专项资金，成立融资租赁投资基金，支持中小企业以融资租赁方式购买设备，把佛山打造成为珠江西岸融资租赁区域中心。实施企业上市“添翼计划”，新增上市企业5家以上、股份制改造企业30家以上。依托广东金融高新区，加快建设千灯湖创投小镇，支持区域股权交易市场发展，打造珠江西岸创投中心。

提高现代服务业发展水平。大力发展商贸会展、信息服务、现代物流等生产性服务业，加快发展育幼、教育、养老健康、休闲娱乐、文化旅游等生活性服务业。发挥广东工业设计城示范带动作用，推进陶谷小镇清华美院设计研究所等项目建设，着力打造“世界设计之都”。以建设3大类综合影视基地、5个特色影视产业聚集区、若干个影视特色衍生产业群、1条影视主题全域黄金旅游线路[]为抓手，加快建设中国南方影视中心，促进影视产业跨越发展。

三、全力推进粤港澳大湾区建设，加快形成全面开放新格局。编制实施大湾区建设实施方案和专项规划，携手大湾区各城市共建国际一流湾区和世界级城市群，努力在更高水平上扩大开放。

全面深化广佛同城。加快推进广佛同城化合作示范区建设，推动三山新城与广州南站接壤片区对接，谋划建设大型城市综合体，打造大湾区标志性项目。推进广佛创新同城，提高产学研协同创新和成果转化水平，共建国际科技创新中心。深化产业协同发展，聚焦先进装备制造、汽车、新一代信息技术、生物医药与健康等产业，共建若干个万亿级产业集群。优化同城化交通网络，加快广佛核心区“两高两快五主干”快速通道[]以及广佛新干线快速化改造、珠江大桥放射线接广佛新干线、番海大桥等项目建设，推进南海新交通与广州南站衔接，争取广州地铁28号线接入佛山西站，共建共享国际空港海港枢纽。深化教育医疗、社会保障、社会治理、污染防治合作，共建广佛优质生活圈。

加强与珠江东西两岸城市合作。深化与珠江东西两岸城市在基础设施建设、产业协同发展、公共服务供给等领域合作对接，加快打造覆盖全市、通达湾区、联络世界的现代化综合交通体系，共同推动先进装备制造产业带与电子信息产业带融合发展。加强与深圳在科技创新、产业协同等领域合作，着力引进电子信息、高端装备制造等深圳产业外溢项目。全面落实“香港+佛山”合作机制，办好第四届“香港·佛山节”，力争开通佛山西站至香港的高铁线路，构建香港—佛山1小时经济圈。建立健全“澳门+佛山”合作机制，加强经贸、文化、金融、中医药等领域合作。高标准规划建设粤港澳合作高端服务示范区，打造粤港澳服务贸易自由化示范基地。

深度参与“一带一路”建设。加强国际产能合作，支持优势行业企业抱团到“一带一路”沿线国家参与经贸园区建设、开展并购和投资设厂。鼓励“走出去”企业返程投资，加快引进国外资金、技术、人才和管理经验。深耕欧美市场，积极开拓东南亚、中东、非洲、东欧等市场，举办多场“佛货全球行”展会，继续推进佛山泛家居品牌产品海外展示体验馆建设，着力打造世界“泛家居商品采购之都”。复制推广自贸试验区改革创新经验，争取广东自贸试验区扩区延伸至佛山。充分发挥中德工业服务区作用，依托中德工业城市联盟，强化对德对欧经贸往来和技术合作。发挥地处珠三角腹地区位优势，积极参与推进广佛肇清云韶经济圈建设，加快粤桂黔高铁经济带合作试验区（广东园）核心区建设，着力把佛山打造成为珠江西岸经贸中心和辐射粤西及大西南的桥头堡。

四、加快集聚创新资源，不断壮大发展新动能。充分发挥创新对建设现代化经济体系的战略支撑作用，加快集聚创新资源和新旧动能转换，努力建设面向全球的国家制造业创新中心。

强化高端创新平台建设。推进三龙湾高端创新集聚区规划建设，加快布局高端高新产业，努力将三龙湾建设成为引领创新发展的重大引擎。实施“一环创新圈”战略规划，打造“东北—科创环”“西南—智造环”，构建“一核五平台多节点”创新体系[]，对接广深港澳科技创新走廊。推动佛山国家高新区聚焦高新技术企业培育和新兴产业集聚，着力建设成为创新发展示范区，力争到2020年跻身全国高新区20强。推动基础研究、应用研究和产业化融通发展，建立健全季华实验室管理体制和运行机制，力争到2020年完成基础设施建设，努力成为国家实验室“预备队”重要一员。深化与中科院、清华大学、北京大学、复旦大学、北京理工大学、中山大学、华南理工大学等科研院校合作，力争新引进大院大所组建新型研发机构10家以上，到2020年累计引进各类型创新平台100家，在关键共性技术、前沿引领技术和颠覆性技术创新攻关上取得突破。发挥佛山绿色发展创新研究院平台作用，努力建设世界级氢能与燃料电池技术研发创新基地。

提升企业自主创新能力。坚持企业是创新的主体，强化分类指导，培育一批具有自主知识产权和核心竞争力的高新技术企业。大力实施高新技术企业树标提质行动计划，力争国家高新技术企业达5000家，培育规模以上标杆高新技术企业50家。支持龙头企业参与国家和省重大创新平台建设，引导中小企业普遍建立研发机构，规模以上工业企业研发机构建有率提高到55%。落实研发费用加计扣除等普惠性政策，引导企业加大研发投入，增强技术创新能力。

推动军民融合发展。以创建国家军民融合创新示范区为突破口，加快形成全要素、多领域、高效益的军民融合深度发展格局。加强与中国电子科技集团等大型军工央企合作，着力引进军民融合重大项目和龙头企业，积极培育智能装备制造、智能穿戴制造、生物智能制造、新一代信息技术、航空航天、新材料和新能源等6大产业，推动军民融合产业集聚发展。加快广东国防科技工业技术成果产业化应用推广中心、中国空间技术研究院佛山军民融合技术推广中心、广东空间网络工程技术研究中心等平台建设，实施一批军民技术重点领域攻关项目，促进军民技术和成果双向转化，争当全国军民科技协同创新引领区。

打造创新人才高地。完善人才扶持政策，通过“靶向引才”“以才引才”“柔性引才”等方式，加快集聚一批“高精尖缺”人才团队和重点产业专业技术人员，新增省级创新创业团队5个、市级创新创业团队40个、中高端人才1500人以上。推行“优粤佛山卡”，为持卡人才提供安居保障、医疗健康、子女入学、金融支持等优质服务。打造南方高等教育名城，加快筹建佛山理工大学，继续推进佛山科学技术学院建设高水平理工科大学，加快建设东北大学佛山研究生院、华南师范大学国际联合学院、广东工业大学佛山研究生院。提升现代职业教育水平，支持佛山职业技术学院、顺德职业技术学院建设一流高职院校，大力培育精准对接产业需求的专业技能人才。

营造创新创业良好生态。完善创新激励机制，赋予科研人员更多的人财物自主支配权。健全孵化育成体系，各类孵化载体增至90家，为企业提供“众创空间+孵化器+加速器+产业园区”全链条服务。加快建设广东高校科技成果转化中心，争取一批国家和省重大项目在佛山开展延展性研究和产业化应用，打通科技成果转化“最后一公里”。发挥中国（佛山）知识产权保护中心、广州知识产权法院巡回法庭作用，加快构建知识产权创造、保护、运用、服务体系。引导企业积极申请国际专利，强化海外商标注册保护，为企业“走出去”营造公平竞争市场环境。

五、加大城乡区域统筹发展力度，提高发展平衡性和协调性。树立全市“一盘棋”思想，着力破解城乡之间、区域之间发展不平衡不充分问题，塑造要素有序自由流动、基本公共服务均等、资源环境可承载、城乡一体化发展的区域协调发展新格局。

强化全市统筹协调发展。坚持以高水平规划引领城市现代化，以城市现代化促进产业高端化，努力实现以城促产、以产兴城。优化国土空间规划编制体系，建立“多规合一”的“一张图一平台”[]，推进城市总体规划修编和空间规划编制，合理配置市域各类空间资源。发挥南海区和顺德区的牵引力、高明区和三水区的加速力以及禅城区的融合力作用，推动“强中心”由“禅桂新”全面拓展为“禅南顺”，统筹产业资源、基础设施、公共服务等适度向高明区、三水区倾斜，逐步缩小各区之间发展差距。

建设现代化基础设施体系。按照适度超前、互联互通、安全高效原则，建设一批重大基础设施工程。加快推进地铁2号线一期、3号线、广州地铁7号线西延顺德段等项目建设，完善佛山西站、三水南站等枢纽建设，继续做好2号线二期、4号线、9号线、11号线和13号线等前期工作。加快“一环”西拓工程建设，基本完成“一环”辅道改造，再打通20条“断头路”。做好珠三角枢纽（广州新）机场前期工作，加快规划临空经济区，积极参与世界级机场群建设。大力发展水路运输，强化岸线资源整合，加快推进佛山新港和澜石港关停，以及三水港区、高明港区扩容，融入粤港澳大湾区港口群。积极创建全国公交都市示范城市，实现全市公交TC管理[]体制一体化，加强公交场站设施建设，加快实现中心城区公交专用道成环成网。全面推广新能源汽车在公共交通领域应用，加大充电桩、加氢站建设力度。积极谋划布局5G网络建设，推动移动通信和宽带网络深度覆盖。实施智能电网三年行动计划，加快凤城输变电等重点电网工程建设。加强海绵城市建设，新建地下综合管廊10公里，推进老城区雨污分流改造。推进绿色建筑扩面提质，新建绿色建筑1000万平方米。创建省装配式建筑示范城市，培育引进一批装配式建筑企业。

提高城市治理精细化水平。全面完成城市治理三年行动计划。推进中心城区形态提升，加快打造“两大滨水核心、一条城市中轴、八大功能节点、多条门户走廊”，推动中心城区面貌“一年一小变、三年大变样”。完善城市更新体制机制，以“绣花”功夫提升城市精细化管理水平。统筹推进“三旧”改造，促进城市“旧貌换新颜”。推进首批市级特色小镇建设，谋划创建第二批市级特色小镇。加强交通拥堵治理，规范共享单车有序发展，引导市民绿色出行。开展“五位一体”沿街景观整治[]，全面治理违法建设和“脏乱差”等现象，实现城市管理考评村居全覆盖。推进城乡生活垃圾分流分类减量，到2020年基本建立生活垃圾分类制度。深入实施“厕所革命”行动，到2020年打造100座示范文明公厕。

强力实施乡村振兴战略。坚持农业农村优先发展，全面推进19类90项乡村振兴重点任务，带动乡村基础设施、公共服务、产业发展、社会治理与城市深度融合。启动一批乡村振兴示范村建设和第四批10个古村落活化升级，深入开展高明革命老区特别帮扶。开展“千村示范、万村整治”工程，全域推进生态宜居美丽乡村建设，争创第三、四批省级新农村示范片。统筹开展农村集体产权制度改革国家整市试点，推动集体资产“应上必上”平台公开交易。加快建设现代农业产业体系。推进省农业科技示范市建设，加强“菜篮子”工程建设，培育农民合作社、家庭农场等新型经营主体，创建一批省级现代农业产业园，增加优质绿色农产品供给。积极发展民宿经济、农村养老服务、农村文化创意、田园综合体等新业态。

六、加快关键环节和重点领域改革，进一步增强经济发展内生动力。抓好改革举措的协同配套、同向共进，将改革进行到底，为高质量发展提供坚实体制机制保障。

全面完成政府机构改革。调整优化政府机构设置和职能配置，合理划分事权，理顺权责关系，构建系统完备、科学规范、运行高效的机构职能体系。出台实施部门“三定”规定，稳步推进区级机构改革和镇（街）机构改革，确保各级机构、职责、人员及时有序调整到位，各项业务无缝衔接、平稳运行。深化事业单位改革，做好本次机构改革后事业单位同步调整工作，推进政事分开、事企分开、管办分离。

加快构建“强市、活区、实镇”发展新格局。深化行政管理体制机制改革，加强市级在规划布局、重大平台、基础设施等方面统筹力度，强化区级政府推动经济发展、城市建设管理和发展社会民生事业等职能，把镇（街）工作重心转到基层治理和公共服务、公共管理、公共安全上来。落实加强市级财政统筹机制工作方案，建立全市性重大项目建设市级财政统筹制度，增强市级对重要战略资源、重大项目、重点工作的统筹力和执行力。按照“市统筹、区建设、市区联动”原则，加快建立健全三龙湾高端创新集聚区管理体制机制，着力打造深化改革开放、强化市级统筹的示范区。优化佛山国家高新区管理体制机制，加强对各园区产业规划、科技创新、招商选资等领域统筹协调力度，进一步释放发展活力。支持顺德区率先建设省高质量发展体制机制改革创新实验区，三水区推进省乡村振兴综合改革试点。深化综合行政执法改革，整合同一领域或相近领域执法队伍，实行综合设置，推动执法力量向基层和一线倾斜。

深入推进“放管服”改革。积极对接国际高标准营商规则体系，加快创建全国社会信用体系建设示范城市，着力建设国际化法治化便利化营商环境。深化行政审批制度改革，再取消调整一批行政许可和中介服务事项。推进工程建设项目审批制度改革，进一步优化业务流程压减审批时限，实现政府投资项目全流程审批时间不超过45个工作日、社会投资项目平均审批时间不超过25个工作日。推进不动产登记能力和作风建设“双提升”，实现抵押登记1个工作日内、其他登记类型3个工作日内办结。推进权责清单标准化工作，压减权责清单事项数量。全面实施市场准入负面清单制度，提升市场事前事中事后监管水平。深化商事制度改革，大力推行准入即准营“证照联办”，构建“一窗受理、联合审批、同步发证”涉企证照审批新模式。提高“数字政府”建设水平，完善“一门式一网式”政府服务模式改革，拓展政务服务网上办事、移动办事、自助办事范围和深度，构建“互联网+政务服务”新格局。

优化土地管理体制。完善土地利用年度计划指标管理政策，分类管理、高效使用各类用地指标，保障重大项目和平台用地。创新增减挂钩市场化管理方式，推动增减挂钩指标公开交易。研究出台产业发展保护区工业用地提升指导意见，推动低效产业用地整治提升，加大对闲置土地、批而未供土地的处置力度，为实体经济发展拓展土地利用空间。

深化国资国企改革。优化国有资本布局，引导国有资本向公共服务、基础设施建设、现代金融及现代服务业等领域加快集聚。积极发展混合所有制经济，鼓励市属国有企业与行业优势企业交叉持股，全面提升国有资产运营质量。完善企业法人治理结构，深入推进职业经理人制度和外部董事制度试点。优化国资监管职能，加快从“管企业”向“管资本”转变。

七、集中力量攻坚克难，推动三大攻坚战取得决定性进展。坚持问题导向，打好防范化解重大风险、精准脱贫、污染防治三大攻坚战，使全面建成小康社会得到人民认可、经得起历史检验。

着力打好防范化解重大风险攻坚战。严控政府债务风险。按照严控增量、清理存量、尽快化解、确保平稳的原则，积极防范化解地方政府债务和隐性债务风险，确保按计划完成存量隐性债务化解任务。主动防范化解金融风险。严厉打击违法金融活动，规范金融机构经营行为，深入开展互联网金融风险专项整治，维护金融稳定，坚决守住不发生区域性系统性金融风险的底线。发挥总规模100亿元上市企业通济基金作用，纾解上市企业股权质押风险。坚持房子是用来住的、不是用来炒的定位，积极稳妥推进住房租赁试点和集体建设用地建设租赁住房试点，探索建立促进房地产市场平稳健康发展长效机制。

着力打好精准脱贫攻坚战。深入推进云浮、湛江精准扶贫，持续实施打赢脱贫攻坚战三年行动，确保扶贫资源投入和使用更加有效。深化佛山对口帮扶云浮产业共建，做大做强“两园六区”优势产业，着力打造产值超100亿元的共建产业园。积极开展东西部扶贫协作，以深化产业合作、人才支持和劳务协作为重点，做好对口扶贫协作四川凉山州工作。继续推进对口支援西藏墨脱县、新疆伽师县、新疆生产建设兵团41团草湖产业园以及四川甘孜州乡城县、得荣县工作。积极做好对口合作黑龙江双鸭山市工作。发挥“万企帮万村”行动示范效应，鼓励和引导更多企业到贫困地区投资兴业，推动贫困人口增收脱贫。

着力打好污染防治攻坚战。突出打好碧水攻坚战，开展江河湖库“五清”专项行动[]，落实省万里碧道工程和“让广东河更美”大行动，积极推进广佛跨界河流污染治理和城乡黑臭水体整治，力争12个省控及以上断面地表水水质优良比例达83.3％，8条城市建成区黑臭水体消除黑臭。实施排水管理条例，加快建设分散式污水处理设施和污水管网，新（改）建接通污水管网1000公里。坚决打赢蓝天保卫战，落实大气污染防治强化措施，严控大气污染项目准入，优化产业、能源、交通运输结构，禁止使用高排放非道路移动机械，推进195家市控重点VOCs企业“一企一策”综合整治，确保PM2.5浓度控制在36微克/立方米以内。扎实打好净土防御战，加强土壤污染风险管控，加快建立重点行业企业污染地块清单和优先管控名录。强化危险废物全过程环境监管体系，加快建设生活垃圾、污泥、危险废物等固体废弃物处置设施。深入推进大湾区高品质森林城市建设，启动美丽佛山五年绿化行动计划，全面实施九大重点生态工程[]，加快云东海国家湿地公园、王借岗公园、李家沙湿地公园等一批大型城市绿心项目建设，推进48个河心岛生态修复整治。全面推行排污许可证制度，优化排污权交易制度。加快构建绿色制造体系，推动绿色产品、绿色工厂、绿色园区和绿色供应链全面发展，积极引导清洁能源消费。强化环保宣传教育，提高全社会生态文明素养。做好中央、省环保督察及市生态环境督察后续工作。

八、加强保障和改善民生，加快形成共建共治共享社会治理格局。坚持以人民为中心的发展思想，精心做好各项民生工作，让群众获得感、幸福感、安全感更加充实、更有保障、更可持续。

优化公共服务供给。健全与经济社会发展水平相适应的基本公共服务统筹机制，稳步推进城镇基本公共服务常住人口全覆盖。实施就业优先政策，推进创业孵化基地群和公共就业人才服务平台建设，实现更高质量和更充分就业。创建国家教育综合改革试验区，加快高水平教育现代化，着力构建以普惠性资源为主体的学前教育公共服务体系，力争完成新（改、扩）建幼儿园44所，新增学位1.8万个，促进普通高中优质特色多样发展。完善养老服务体系，取消养老机构设立许可，扶持民办养老机构发展，加快建设市颐养院和新社会福利院。深化基本医疗保险城乡一体化改革，推进基本医疗保险支付方式改革。探索政府引导、社会力量筹资、慈善组织运作的政社联动救助模式，支持禅城区开展全国社会救助综合改革试点。深化医药卫生体制综合改革，推进紧密型医联体建设，促进优质医疗资源往基层下沉。深入实施“登峰计划”，力争到2022年3家重点建设医院跻身全国同类医院百强，4家培育建设医院排名全省同类医院前五位。加快建设中医药强市，积极创建全国基层中医药工作先进单位。建立健全退役军人服务保障体系，完善退役军人服务管理、就业创业、优先优待等政策措施，做好优抚安置工作，争创全国双拥模范城“九连冠”。开展新市民融入行动计划，实施新市民积分制服务管理新办法，有序解决新市民户籍、住房、教育等问题，促进公共服务资源公平共享。

推进文化导向型城市建设。提升公共文化设施服务效能，巩固国家公共文化服务体系示范区创建成果。强化历史文化资源保护、开发和利用，挖掘南粤古驿道历史文化基因，办好广东非遗周暨秋色巡游活动，加快建设市博物馆和粤剧文化园，积极推进“博物馆之城”“世界美食之都”“世界功夫之城”建设。繁荣文学艺术创作，围绕庆祝新中国成立70周年组织开展系列文化活动，打造系列文艺精品。巩固全国文明城市成果，打造“乐善之城”“敬业之城”“志愿者之城”。深入开展全民健身活动，推进社区体育公园等公共体育场地设施建设。办好篮球世界杯（佛山赛区）比赛、定向世界杯决赛、国际龙舟赛等赛事活动。

加强和创新社会治理。全面推进社区“五化”建设[]，统筹推进“网格化”“社区通”“村务通”等平台建设，打造社会综合治理云平台。完善非户籍常住人口参与基层治理机制，50%社区建立社会组织联合会。加强村级组织标准化建设，制定村民委员会、村民小组、村务监督委员会等组织建设标准，提升基层自治组织服务能力和基层善治水平。扶持社工人才队伍和志愿服务组织建设，推行“三社联动”[]社区服务模式。发挥普法宣传、人民调解和律师队伍作用，深化“一村居一法律顾问”工作，完善四级公共法律服务网络。健全社会矛盾纠纷排查化解机制，构建群众诉求服务体系，推动信访矛盾化解。

深入推进平安佛山建设。持续开展扫黑除恶专项斗争，积极推进全国禁毒示范城市创建，筑牢立体化社会治安防控体系，把佛山打造成为全国最安全稳定、最公平公正、法治环境最好的城市之一。加强应急管理工作，健全安全生产责任体系，建设智慧安全城市，深入开展危险化学品、交通运输、建筑施工等重点行业领域安全生产综合治理，持续推进“智能制造、本质安全”示范创建、国际（国家）安全社区创建等活动。创建国家食品安全示范城市，推进食品药品安全三年升级计划，构建公平公开的食品药品生产经营环境和共治共享的食品安全社会治理格局。推进“南粤粮安工程”，保障粮食安全。

扎实办好十件民生实事。一是城乡居民养老保险基础养老金提高至每人每月260元。二是新（改、扩）建义务教育学校30所，新增义务教育阶段学位3.1万个。三是推进普惠性幼儿园生均拨款按星级分类扶持。四是市妇女儿童医院建成投入使用。五是免费筛查新生儿疾病，综合防控儿童青少年近视。六是完成省下达的住房保障工作目标任务。七是提高低保和特困人员供养标准。八是开展文化进园区普惠性活动1000场（次）。九是新增（改造）公园绿地面积81公顷。十是建设20项民生水利重点工程。

九、加强政府自身建设，努力建设人民满意的服务型政府。坚持和加强党的全面领导，加快政府职能转变，切实增强履职能力，着力提升政府治理体系和治理能力现代化水平。

加强政治建设。切实提高政治站位，树牢“四个意识”，坚定“四个自信”，坚决做到“两个维护”，自觉在思想上政治上行动上同以习近平同志为核心的党中央保持高度一致。严格落实全面从严治党主体责任，严明政治纪律和政治规矩，以良好的政治文化涵养风清气正的政治生态。严格执行中央八项规定及其实施细则精神，落实党风廉政建设各项规定，深化廉政风险防控，永葆为民务实清廉的政治本色。

加强法治政府建设。严格遵守宪法和法律，运用法治思维和法治方式开展工作，确保政府工作在法治轨道上运行。认真执行人大及其常委会的决议决定，自觉接受人大的法律监督和工作监督，主动接受人民政协的民主监督，广泛听取各民主党派、工商联、无党派人士和人民团体意见，全面接受司法监督、群众监督和舆论监督，自觉接受巡视监督和监察委员会的监督，推进审计监督全覆盖。遵守法定权限和程序，严格规范公正文明执法，做好行政复议工作。加大政务公开力度，着力构建决策科学、执行坚决、监督有力的权力运行机制，持续提升政府公信力。

加强作风建设。把雷厉风行和久久为功结合起来，全面整治“四风”问题特别是形式主义、官僚主义以及庸政懒政怠政，完善政绩考核评价机制，压实工作责任，确保各项决策部署落到实处。加强和改进调查研究，认真解决群众和企业反映强烈的突出问题，推进服务型政府建设。严格规范督查检查考核事项，落实激励机制和容错纠错机制，营造崇尚实干的良好氛围。坚持厉行节约，一般性支出按照不低于5%的比例压减，严控“三公”经费预算。加强公务员队伍建设，提高科学决策水平和精准施策水平，打造忠诚干净担当的高素质公务员队伍。

各位代表！乘风好破浪，奋进正当时。让我们更加紧密地团结在以习近平同志为核心的党中央周围，高举习近平新时代中国特色社会主义思想伟大旗帜，在省委、省政府和市委的坚强领导下，不忘初心、牢记使命，锐意进取、奋勇争先，努力在新时代展现新作为、创造新业绩，为广东实现“四个走在全国前列”、当好“两个重要窗口”作出新的更大贡献！
